# Evaluation of hyperprogressive disease with atezolizumab plus bevacizumab for hepatocellular carcinoma: A secondary analysis of the IMbrave150 trial

**DOI:** 10.1002/ijc.35407

**Published:** 2025-03-13

**Authors:** Yuan Gao, Ann‐Lii Cheng, Lee X. Li, Natalie Parent, Ganessan Kichenadasse, Christos S. Karapetis, Andrew Rowland, Ashley M. Hopkins, Michael J. Sorich

**Affiliations:** ^1^ College of Medicine and Public Health Flinders University Adelaide South Australia Australia; ^2^ National Taiwan University Cancer Center Taipei Taiwan; ^3^ Department of Medical Oncology Flinders Medical Centre Adelaide Australia

**Keywords:** combination immunotherapy, hepatocellular carcinoma, hyperprogressive disease, immune checkpoint inhibitor, PD‐1 blockade

## Abstract

The use of Immune checkpoint inhibitors (ICIs) as monotherapy for patients with hepatocellular carcinoma (HCC) has been associated with an increased risk of hyperprogressive disease (HPD), the occurrence of which carries a poor prognosis. However, it is unknown whether contemporary frontline treatment with the combination of atezolizumab and bevacizumab causes significant HPD. This study conducted a secondary analysis of patient‐level data from the IMbrave150 randomized controlled trial of atezolizumab plus bevacizumab versus sorafenib for frontline treatment of HCC. Multiple established definitions of early progression and treatment failure applicable to clinical trials were evaluated, including Response Evaluation Criteria in Solid Tumours (RECIST) HPD, HPD based on percent change of sum of longest diameter (SLD HPD), treatment failure HPD (TF HPD), and fast progression (FP). The incidence of these measures was compared between arms. The risk factors for and prognosis of TF HPD were evaluated. The risk of RECIST HPD and TF HPD was significantly lower with atezolizumab plus bevacizumab treatment than with sorafenib treatment—odds ratio for RECIST HPD: 0.29 (95% CI 0.01 to 0.82), TF HPD: 0.30 (0.17, 0.54). TF HPD was similarly associated with poor prognosis, irrespective of treatment arm. High blood alpha‐fetoprotein and neutrophil‐to‐lymphocyte ratio were both associated with an increased risk of TF HPD. For all definitions of early progression/treatment failure, the risk was either significantly lower with atezolizumab plus bevacizumab than with sorafenib, or there were no differences. Atezolizumab plus bevacizumab treatment is unlikely to cause significant HPD.

## Graphical abstract

What's new?

Hyperprogressive disease (HPD) is defined by unusually rapid tumor growth following treatment initiation. In hepatocellular carcinoma (HCC), HPD is suspected of occurring more commonly with immune checkpoint inhibitors (ICIs), particularly when used as monotherapy, than with other treatment strategies. Here, combination treatment using the ICI atezolizumab plus the antiangiogenic agent bevacizumab was investigated for associations with HPD in HCC patients. Clinical data show that the combination is unlikely to increase HPD risk over sorafenib, a previous standard‐of‐care treatment for HCC. HPD detected in patients administered the combination treatment may be part of the HCC disease course.

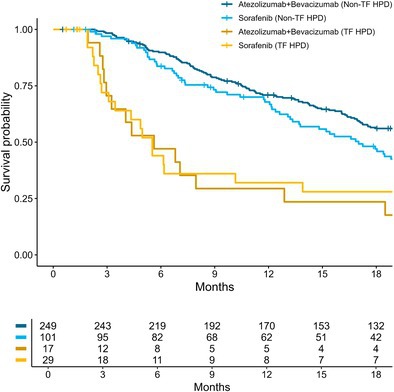

**Abbreviations**
AFP: alpha‐fetoprotein
ALBI: Albumin‐Bilirubin Grade
CI: confidence interval
CRP: C‐reactive protein
HCC: hepatocellular carcinoma
HPD: hyperprogressive disease
ICI: immune checkpoint inhibitors
LMR: lymphocyte to monocyte ratio
NLR: neutrophil‐to‐lymphocyte ratio
OR: odds ratio
OS: overall survival
PD‐1/PD‐L1: programmed cell death protein 1/programmed death‐ligand 1
PLR: platelet to lymphocyte ratio
PLR: platelet to lymphocyte ratio
RCT: randomized controlled trial
RECIST: Response Evaluation Criteria in Solid Tumors
VEGF: vascular endothelial growth factor

## 1 INTRODUCTION

Liver cancer is one of the leading causes of cancer death worldwide, and hepatocellular carcinoma (HCC) is its most common form.^1^ Immune checkpoint inhibitors (ICIs) are an important class of treatments that have significantly improved survival outcomes across many cancer types.^2^ Although ICI monotherapy failed to establish overall survival (OS) benefits in HCC,^3^ the IMbrave150 trial demonstrated that frontline use of the ICI atezolizumab in combination with bevacizumab (an anti‐vascular endothelial growth factor [VEGF]‐targeted therapy) conferred superior OS to the previous standard of care (sorafenib).^4^ Based on these results, atezolizumab plus bevacizumab is a first‐line treatment option for unresectable HCC.^5^, ^6^, ^7^, ^8^

ICI therapy has been associated with a risk of atypical response patterns.^9^ Hyperprogressive disease (HPD) is characterized by accelerated tumor growth following the commencement of treatment and has been associated with poor survival outcomes.^10^, ^11^, ^12^, ^13^, ^14^ The incidence of HPD following the use of ICI therapy is reported to vary across cancers and may differ by treatment modality, such as ICI mono‐ versus combination therapy.^9^, ^15^, ^16^, ^17^, ^18^ Existing evidence supporting the risk of HPD with ICI use is limited by the variable definitions of HPD and the relatively minimal evaluation of HPD following contemporary ICI combination therapies.^19^, ^20^, ^21^ Additionally, very few studies have included the evaluation of a control treatment cohort—without which it is very difficult to separate HPD that is part of the natural history of disease from HPD that is caused by ICI therapy specifically. The optimal approach to detect the causal effect of ICI therapy on HPD is by conducting a randomized controlled trial (RCT), where ICI/non‐ICI populations are exchangeable. However, the most prevalent definitions of HPD, recognized as tumor growth kinetics/rates (TGK/TGR),^10^, ^22^, ^23^ prevent the utilization of data from RCT as it requires pre‐treatment tumor scanning. To overcome this issue, RECIST‐based criteria,^20^, ^24^ as well as other surrogates such as “fast progression”^25^ have been developed for use with RCT data.

Relatively little is known about the risk of HPD with ICI use in advanced HCC.^9^, ^15^, ^16^, ^17^, ^18^ Previous studies have been limited to relatively small observational retrospective analyses that predominantly evaluated ICI monotherapy.^9^, ^15^, ^16^, ^17^, ^18^ Only one study included a (non‐randomized) treatment comparison cohort. This study of predominantly pretreated patients reported an HPD incidence of 13% following treatment with nivolumab monotherapy compared to 0% following treatment with regorafenib or best supportive care.^9^ Whether this marked increase in HPD with ICI monotherapy is also present with contemporary frontline combination ICI therapy is unknown, and it has been speculated that the use of VEGF‐targeted therapy (e.g., bevacizumab) may mitigate the risk of HPD.^9^, ^22^

This study aimed to evaluate the risk of HPD with atezolizumab plus bevacizumab compared to sorafenib for patients with unresectable HCC in the IMbrave150 randomized controlled trial (RCT). Secondary aims were to evaluate the prognosis associated with HPD and to identify risk factors for HPD.

## 2 METHODS

IMbrave150 was a randomized, open‐label, phase 3 trial on the efficacy and safety of atezolizumab plus bevacizumab as the first‐line systemic treatment of locally advanced metastatic or unresectable (or both) HCC.^4^ Patients were randomly assigned in a 2:1 ratio to receive atezolizumab plus bevacizumab or sorafenib.^4^ Blinded independent review of tumor imaging based on Response Evaluation Criteria in Solid Tumours (RECIST) v1.1 criteria was undertaken at baseline and every 6 weeks until week 54 (and subsequently every 9 weeks). This secondary analysis excluded patients treated in mainland China due to privacy laws restricting health data sharing.^26^

### 2.1 Definition of HPD

There is no consensus on a single HPD definition, particularly one applicable to clinical trial data. This study evaluated multiple established definitions of HPD applicable to clinical trials based on measures of early tumor progression and early treatment failure.

Matos and colleagues defined HPD based on RECIST criteria (RECIST HPD). RESCIST HPD was defined as (i) a ≥10 mm increase in the sum of longest diameters (SLD) of target lesions at week 6 compared to baseline and (ii) plus either a ≥40% increase in SLD of target lesions at week 6, or a ≥20% increase in SLD of target lesion with 2 or more new lesions in different organs at week 6.^19^, ^24^, ^27^

Yoon‐Koo et al. defined HPD based on SLD change in target lesions (SLD HPD).^20^ SLD HPD was defined by the percent increase in SLD of target lesions at thresholds of 20%, 40%, and 100% for the first on‐treatment scan (week 6 in our data).

Kim and colleagues defined HPD based on early treatment failure (TF HPD).^10^, ^14^ TF HPD was defined as treatment discontinuation due to disease progression or death within 8 weeks after treatment initiation.

Additionally, this study evaluated fast progression (FP) by Gandara et al.^25^ FP is identified as a 50% increase in SLD of target lesions at week six, or death due to disease progression without radiographic assessments within 12 weeks of treatment initiation. If one dies within 12 weeks with a radiographic assessment, FP is defined by the scanning result rather than death.

For subsequent exploratory analyses of predictors of HPD and the association between HPD and survival outcomes, TF HPD (by Kim et al.)^10^, ^14^ was utilized based on having the greatest statistical power.

### 2.2 Predictors of HPD

Baseline variables evaluated as potential predictors of HPD were selected based on prior research investigating the occurrence of HPD or prognosis of patients with HCC.^15^, ^16^, ^19^ Evaluated predictors included age, neutrophil‐to‐lymphocyte ratio (NLR), number of metastatic sites, Child‐Pugh class, alpha‐fetoprotein level (AFP), Albumin‐Bilirubin Grade (ALBI), platelet to lymphocyte ratio (PLR), c‐reactive protein (CRP), and lymphocyte to monocyte ratio (LMR). AFP was log transformed due to the skewed distribution. Laboratory biomarkers were selected on the basis of reported association with HPD^9^ or survival outcome for patients receiving ICI treatment.^28^ ALBI grade was categorized from the ALBI score with three levels, Grade 1 (≤−2.60), Grade 2 (−2.60 to −1.39), and Grade 3 (>−1.39).^29^

### 2.3 Statistical analysis

The frequency and proportion of HPD events were estimated for each treatment arm. Logistic regression was used to quantify the difference in HPD risk between atezolizumab plus bevacizumab treatment and sorafenib treatment. The difference in HPD risk between treatments was reported as an odds ratio (OR) with a 95‐percent confidence interval (95%CI).

A multivariable logistic regression model to predict TF HPD was developed using stepwise inclusion of predictors based on the Akaike Information Criterion (AIC). Predictor associations with TF HPD were reported in terms of the odds ratio (OR) with a 95%CI. Exploratory analysis was conducted for the biomarkers included in the final model. The percent change of these biomarkers from baseline to HPD onset was compared between treatment arms.

A landmark approach was employed to compare the median OS for HPD/FP and non‐HPD/FP populations by different HPD definitions and treatment arms. The log‐rank test and the Kaplan–Meier estimator were used to evaluate survival differences between treatment arms. All statistical analyses utilized R version 4.2.0.

## 3 RESULTS

### 3.1 Comparison of HPD incidence

Of the 408 patients in the safety population, 408 were evaluable for TF HPD and FP; 375 were evaluable for RECIST HPD and target lesion progression‐HPD based on the availability of tumor size assessment at week 6 (Figure S1 and Table S1). There was no difference between the two treatment groups regarding baseline characteristics, liver function, prior disease, and baseline laboratory values (Tables S1 and S2). The overall incidence of early progression and treatment failure amongst all evaluable patients in the IMbrave150 trial varied by HPD definitions—4.0% (15/375) for RECIST HPD, 13.5% (55/408) for TF HPD, and 3.0% (16/408) for FP. By SLD HPD, 12.3% (46/375) and 3.5% (13/375) of individuals had a 20% and 50% increase in SLD, respectively, at the week 6 assessment.

For most HPD definitions, the risk of early progression and treatment failure was significantly lower with atezolizumab plus bevacizumab than with sorafenib (Table 1). The risk of HPD according to RECIST criteria (by Matos) was 2.3% for patients treated with atezolizumab plus bevacizumab compared to 7.6% for patients treated with sorafenib (*p* = 0.023, Table 1). The risk of TF HPD was 8.5% with the atezolizumab plus bevacizumab arm and 23.5% with the sorafenib arm (*p* = <0.001, Table 1). The risk of SLD HPD was significantly lower for atezolizumab plus bevacizumab at the 50% threshold (1.9% vs. 6.8%) but not the 20% threshold (11.7% vs. 13.6% Table 1). The differences in FP were not statistically significant, but the direction of effect numerically favored the atezolizumab plus bevacizumab arm (2.6% vs. 6.6%, Table 1).

**TABLE 1:** Incidence of early progression and treatment failure according to established definitions for atezolizumab plus bevacizumab and sorafenib‐treated patients.

Definitions	Atezolizumab + bevacizumab	Sorafenib	Odds ratio (95% CI)	*p*
	% (n/N)	% (n/N)		
RECIST HPD (Matos et al.^19^, ^24^, ^27^)^a^ (i) A ≥10 mm increase in the SLD of target lesions at week 6 compared to baseline, AND (ii) either a ≥ 40% increase in SLD of target lesions at week 6, OR a ≥20% increase in SLD of target lesion with 2 or more new lesions in different organs at week 6.	2.3% (6/257)	7.6% (9/118)	0.29 (0.10, 0.82)	0.023
SLD HPD (Yoon‐koo et al.^20^)^a^ Percent increase in SLD of target lesions for the first on treatment scan.				
≥20%	11.7% (30/257)	13.6% (16/118)	0.84 (0.45, 1.65)	0.605
≥50%	1.9% (5/257)	6.8% (8/118)	0.27 (0.08, 0.84)	0.025
≥100%	0% (0/257)	0% (0/118)	NA	NA
Treatment failure HPD (Kim et al.^10^, ^14^) Treatment discontinuous due to disease progression, OR death within 8 weeks after ICI initiation	8.5% (23/272)	23.5% (32/136)	0.30 (0.17, 0.54)	<0.001
Fast progression (Gandara et al.^25^) 50% increase in SLD of target lesions at week 6, OR death due to disease progression without radiographic within 12 weeks since treatment initiation. If one die within 12 weeks with radiographic assessment, FP is defined by the scanning result rather than death.	2.6% (7/272)	6.6% (9/136)	0.37 (0.13, 1.01)	0.056

Abbreviations: CI, confidence interval; HPD, hyperprogressive disease; n, number of subjects experiencing the event; N, number of subjects at risk; NA, not applicable; OR, odds ratio; SLD, sum of the longest diameter.

^a^ Excludes subjects who did not have an eligible SLD assessment.

### 3.2 Comparison of prognosis and HPD risk predictors

**FIGURE 1:**
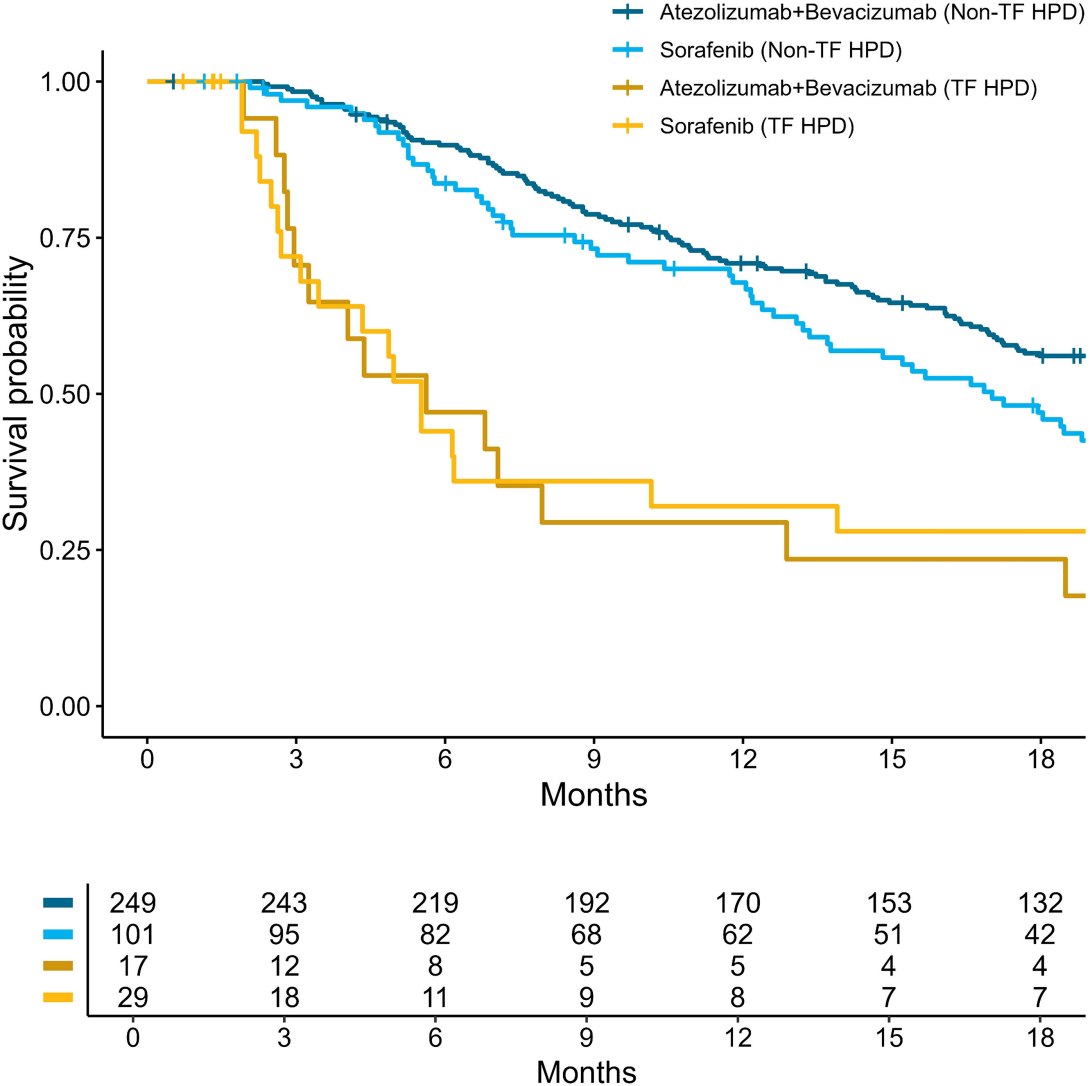
Overall survival by treatment failure HPD (defined by Kim et al.^12^, ^16^) and randomized treatment arms.

Overall, patients identified as having HPD/FP had markedly unfavorable OS prognosis compared to patients without HPD/FP (Table 2, Figure 1). No statistically significant difference in OS was observed between the atezolizumab plus bevacizumab arm and the sorafenib arm for patients who experienced HPD/FP (Table 2 and Figure 1). Notably, for patients not experiencing HPD/FP, subsequent OS was significantly more favorable for patients treated with atezolizumab plus bevacizumab than for those treated with sorafenib (Table 2 and Figure 1).

**TABLE 2:** Median OS (months) according to established definitions for early progression and treatment failure by treatment arms.

Definitions	HPD/FP population			*p* ^a^	Non‐HPD/FP population			*p* ^a^
	Overall	Atezolizumab + bevacizumab	Sorafenib		Overall	Atezolizumab + bevacizumab	Sorafenib	
RECIST HPD (Matos et al.^19^, ^24^, ^27^)^b^, ^c^ *(week 6 landmark)*	4.34	3.68	10.15	0.453	18.40	20.4	14.8	0.005
SLD HPD (Yoon‐koo et al.^20^)^b^ *(week 6 landmark)*								
20%^c^	5.57	6.21	5.24	0.739	19.91	23.5	15.2	0.001
50%^c^	5.62	5.62	7.24	0.894	18.04	20.4	13.9	0.005
100%	NA	NA	NA	NA	NA	NA	NA	NA
Treatment failure HPD (Kim et al.^10^, ^14^)^c^ *(week 8 landmark)*	5.52	5.62	5.52	0.764	20.34	22.8	17.0	0.028
Fast progression (Gandara et al.^25^) *(week 12 landmark)*	10.2	10.8	10.2	0.708	19.9	22.8	17.0	0.057

Abbreviations: FP, fast progression; HPD, hyperprogressive disease; SLD, sum of the longest diameter.

^a^ *p* values come from log‐rank test, by comparing overall survival for each definition between treatments, within subgroups of HPD/FP or non‐HPD/FP population.

^b^ Excludes subjects who did not have an eligible SLD assessment.

^c^ Log‐rank test *p* ≤0.05 on overall survival for HPD/FP subgroup vs. Non‐HPD/FP subgroup for each definition.

In the univariable analysis, age, PLR, CRP, LMR, NLR, and level of AFP were found to be associated with HPD (Table 3). NLR, AFP level, and age were selected in the final multivariable model according to the stepwise AIC algorithm, where NLR and AFP were positively associated with HPD, while age exhibited an inverse association.

**TABLE 3:** Predictive factors of treatment failure HPD (defined by Kim et al.^10^, ^14^) based on logistic regression.

Predictors (measured at baseline)	Univariable analysis		Multivariable analysis	
	OR (95% CI)	*p*	OR_adj_ (95% CI)	*p*
Age^a^	0.65 (0.50, 0.85)	0.001	0.72 (0.54, 0.97)	0.032
Platelet to lymphocyte ratio^a^	1.66 (1.27, 2.15)	<0.001		
C‐reactive protein^a^	1.27 (1.07, 1.50)	0.006		
Lymphocyte to monocyte ratio^a^	0.52 (0.33, 0.83)	0.006		
Neutrophil to lymphocyte ratio^a^	1.47 (1.18, 1.83)	<0.001	1.50 (1.16, 1.93)	0.002
Number of metastatic sites				
0	ref			
1	1.66 (0.90, 3.09)	0.105		
2 or more	2.02 (0.70, 5.21)	0.163		
Alpha‐fetoprotein level^a^	2.72 (1.68, 4.41)	<0.001	2.44 (1.44, 4.14)	<0.001
Albumin‐Bilirubin Grade				
1	ref			
2	1.55 (0.87, 2.82)	0.141		
Child‐Pugh score				
5	ref			
6 or 7	0.85 (0.43, 1.60)	0.632		
Treatment arms				
Sorafenib	ref			
Atezolizumab + bevacizumab	0.30 (0.16, 0.54)	<0.001	0.22 (0.11, 0.42)	<0.001

^a^ Display of effect size was based on per 10 years increased age and per interquartile range change (25%–75%) for biomarkers: platelet to lymphocyte ratio (94–192), C‐reactive protein (1.7–18 mg/L), lymphocyte to monocyte ratio (1.8–3.6), neutrophil to lymphocyte ratio (2.0–4.2), alpha‐fetoprotein level (1.8–7.5 μg/L).

Additionally, an increase in blood AFP from baseline to HPD onset/week 8 assessment was observed in the HPD population across both arms (Figure S2). However, there was no statistically significant difference in percent change of NLR or AFP for patients who received sorafenib compared to patients who received atezolizumab plus bevacizumab (Figure S2).

## 4 DISCUSSION

Our study identified a relatively low risk of HPD/FP for patients with unresectable HCC treated with atezolizumab plus bevacizumab—ranging from 3.0% to 13.5%, depending on the definitions. Importantly, for all definitions of HPD, the risk of HPD was significantly lower for patients randomly allocated to atezolizumab plus bevacizumab treatment compared to patients randomly allocated to sorafenib treatment. This indicates that, in contrast to a prior observational study that reported a higher risk of HPD with ICI monotherapy for pre‐treated HCC,^9^ frontline ICI use in combination with VEGF‐targeted therapy appears unlikely to cause excess HPD in HCC.

Prior studies of HPD in HCC have reported an incidence ranging from 8% to 14.5% for ICI monotherapy,^9^, ^15^, ^16^, ^18^, ^30^ and 1.7% to 10.2% for ICI combination therapy (atezolizumab plus bevacizumab).^17^, ^30^, ^31^ The HPD incidence reported here was generally similar to those of prior studies of HCC, which utilized different definitions of HPD. With respect to prior evaluations of HPD based on data from RCTs, similar findings of no increased risk of HPD with ICI therapy were observed in RCTs comparing ICI/chemoimmunotherapy with placebo in gastric cancer and small cell lung cancer.^20^ Further, no difference in risk of HPD between chemoimmunotherapy and chemotherapy alone was identified in the first‐line setting of NSCLC.^19^ Gandara et al., using ‘fast progression’ criteria (50% increase in SLD or early death from treatment initiation within 6 weeks), did not identify any difference between immunotherapy and chemotherapy.^25^

NLR, AFP blood levels, and age were found to be associated with a significantly higher risk of TF HPD, which is concordant with prior studies of HPD risk factors.^9^, ^15^, ^16^, ^17^, ^19^ More generally, NLR has been reported to be a strong prognostic factor for survival outcomes across many advanced cancers.^32^, ^33^, ^34^ As per prior studies,^10^, ^11^, ^12^, ^13^, ^14^ individuals with HPD defined at the early assessment (weeks 6 to 12) had markedly unfavorable OS compared to individuals without HPD. More generally, early changes in tumor size are prognostic of survival outcomes.^35^ Individuals with HPD following treatment with atezolizumab plus bevacizumab had similar OS to individuals with HPD following treatment with sorafenib—indicating that the prognosis associated with HPD detected is comparable between treatments. This suggests that for frontline ICI combination therapy for HCC, HPD is associated with poorer prognosis and that this HPD likely reflects the natural course of the disease (for a subset of individuals) rather than being a phenomenon that is ICI‐specific.

It has been speculated that the risk of HPD with ICI therapy in HCC may be mitigated by its use in combination with VEGF‐targeted therapy.^9^, ^22^ Based on an observational study of 198 patients with HCC, HPD risk was reported to be lower in patients receiving atezolizumab plus bevacizumab than ICI monotherapy.^30^ Atezolizumab and bevacizumab have different mechanisms of action, where the former blocks PD1/PD‐L1 interaction, therefore, stimulating the T cell immune response to kill cancer cells^36^; the latter targets VEGF, leading to inhibition of neo‐vascularization.^37^ It has been reported that anti‐angiogenesis may assist tumor immune microenvironment reprogramming, converting the tumor microenvironment from immunosuppressive to immunosupportive.^38^ More broadly, it may be speculated that a combination of ICI with a range of other therapies (e.g., chemotherapy) may mitigate the risk of HPD.^19^ Evaluating HPD in the phase 3 HIMALAYA study of durvalumab plus tremelimumab (combination of two different ICIs) vs. sorafenib would provide further insight regarding whether combination therapy generally or only specific combinations may mitigate HPD risk.^39^

The strengths of this study are the high‐quality data and consistent follow‐up from a prospective clinical trial, and specifically the blinded independent assessment of tumor response. In comparison, most prior studies of HPD are based on retrospective analysis of real‐world treatment, which is often associated with substantial heterogeneity in the timing and evaluation of tumor size changes. Additionally, the focus of this study on a contemporary first‐line treatment option for HCC (atezolizumab plus bevacizumab) is also a strength of the study—in contrast to most prior studies of HPD that typically focus on later‐line ICI monotherapy. Finally, a key advantage of this study is the use of a contemporaneous control arm, which was randomly assigned—thereby enabling estimation of outcomes between treatments that are unbiased by confounding. In contrast, most prior evaluations of HPD have not included a control treatment arm, which makes it very difficult to determine whether HPD observed is due to ICI treatment specifically or is simply a natural course of the disease that would have been observed with non‐ICI treatments.

A limitation of this study is that the inclusion and exclusion criteria of the clinical trial may not fully represent the general population treated in routine clinical practice. Although this study excluded the cohort from China, there was a low risk of bias as the study randomization was stratified by geographic region.^4^ Our study evaluated many biomarkers potentially associated with HPD; however, there are other HCC biomarkers that may be associated with HPD, such as vitamin K absence‐II.^28^ Additionally, as previously described, only a single pre‐treatment evaluation of tumor size was available, which prevents estimation of HPD based on changes in tumor growth kinetics/rates. Although the prevalence of HPD varies according to different criteria,^10^, ^24^ the RECIST criteria demonstrated good agreement with TGK/TGR criteria.^24^ Moreover, the study evaluated many different tumor and clinical event indicators of HPD, which demonstrated consistent findings of no increased risk. Additionally, the availability of multiple pre‐treatment measures of lesion size is often limited in the frontline treatment setting—even in retrospective analyses of routine care. Nonetheless, it would be valuable for future trials to collect historical tumor size measurements, such as from clinical cancer centers, to enhance the capability to evaluate tumor growth rate pre‐treatment versus on‐treatment. Furthermore, this study also minimized the chance of misclassification bias by employing multiple HPD definitions and standardized, independent tumor assessments.

An important strength of this study is the evaluation of HPD utilizing data from an RCT that formed the evidence base of the current standard of care. RCT data enables the inclusion of appropriate treatment controls and hence helps to differentiate whether HPD is caused by a specific treatment or it is a natural course that will be observed similarly for all treatments. Additionally, unlike most prior studies of HPD, this study evaluated first‐line therapy, which is particularly relevant to the contemporary use of ICI therapy. Further, this study included clinical criteria of early treatment failure, which is important for avoiding bias that may result from the exclusion of individuals without a post‐treatment tumor measurement.

In conclusion, among unresectable HCC patients in the first‐line systemic treatment setting, there was no indication of any increased risk of HPD with atezolizumab plus bevacizumab compared to sorafenib across multiple definitions and indicators of HPD. Rather, for definitions of HPD, the risk of HPD was significantly lower with atezolizumab plus bevacizumab than with sorafenib. The HPD detected, therefore, likely reflects the natural course of the disease for a subset of individuals. The HPD subpopulation had unfavorable survival outcomes, and high NLR and high AFP levels were associated with an increased risk of HPD.

## AUTHOR CONTRIBUTIONS

**Yuan Gao:** Conceptualization; writing – original draft; methodology; writing – review and editing; formal analysis; software; data curation; visualization. **Ann‐Lii Cheng:** Writing – review and editing. **Lee X. Li:** Writing – review and editing. **Natalie Parent:** Validation; writing – review and editing. **Ganessan Kichenadasse:** Writing – review and editing. **Christos S. Karapetis:** Writing – review and editing. **Andrew Rowland:** Writing – review and editing. **Ashley M. Hopkins:** Funding acquisition; supervision; resources; writing – review and editing; conceptualization; methodology. **Michael J. Sorich:** Conceptualization; funding acquisition; supervision; resources; methodology; writing – review and editing.

## FUNDING INFORMATION

The project was supported by a research grant (GNT2013565) from Australia's National Health and Medical Research Council (NHMRC). Michael J. Sorich is supported by a Beat Cancer Principal Research Fellowship from the Cancer Council of South Australia. Ashley M. Hopkins is supported by an NHMRC Investigator Fellowship (APP2008119).

## CONFLICT OF INTEREST STATEMENT

Yuan Gao, Lee X. Li, Natalie Parent, Ganessan Kichenadasse have nothing to disclose. Irrelevant to this study, Ann‐Lii Cheng received research funding from F. Hoffmann‐La Roche Ltd. to the institution. Ann‐Lii Cheng also received a consulting fee and/or payment for an educational event from Eisai, Ono Pharmaceutical, Ipsen Innovation, Bayer Healthcare, Merck Sharp and Dohme, Chugai Pharmaceutical, Novartis, and Amgen Taiwan, while these were irrelevant to this study. Outside of this study, Ann‐Lii Cheng also participates in a data safety monitoring board for Novotech. Outside of this study, Christos S Karapetis receives consulting fees and/or payment for educational events from Eisai, Merck Sharp and Dohme, Takeda, Beigene, Bristol myers squibb, Boehringer Ingelheim, Pierre Fabre, Eli Lilly, and Servier. Without payment, he served as a chair member of the Breast Cancer Trials Australia, IDSMC, and the chair of the Scientific Advisory Committee from the Australasian Gastrointestinal Trials Group. Outside of this study, Michael J. Sorich and Andrew Rowland received funding from Pfizer and Boehringer Ingelheim to the institution. Outside of this study, Ashley M. Hopkins received funding from Tour De Cure and The Hospital Research Foundation. Andrew Rowland received payment for an educational event from Boehringer Ingelheim and Genentech, but these were irrelevant to this study. Andrew Rowland is the director of the Australian New Zealand Society of Pharmacologists and Clinical Toxicologists. Ashley M. Hopkins was supported by NHMRC Investigator grant to conduct this study.

## ETHICS STATEMENT

Ethics approval statement and patients consent statement refer to the original clinical trial (IMBRAVE150; clinicalTrials.gov number: NCT03434379). This study is based on secondary analysis from the clinical trial; therefore, it is not applicable.

## Supporting information


**DATA S1.** Supporting Information.

## Data Availability

This publication is based on research using data from Roche that has been made available through Vivli, Inc. Vivli has not contributed to or approved, and is not in any way responsible for the contents of this publication. Further details and other data that support the findings of this study are available from the corresponding authors upon request. The processes for assessing eligibility for IPD sharing and requesting access to clinical trial data have previously been described in detail by our research team.^40^, ^41^
