# Supplementary material for: Evaluation of hyperprogressive disease with atezolizumab plus bevacizumab for hepatocellular carcinoma: A secondary analysis of the IMbrave150 trial
Source: Int J Cancer. 2025 Mar 13;157(2):336–44. doi: 10.1002/ijc.35407 (PMC12079626; doi:10.1002/ijc.35407)

Evaluation of hyperprogressive disease with atezolizumab plus bevacizumab for hepatocellular carcinoma: a secondary analysis of the IMbrave150 trial

(Supplementary documents)

Authors: Yuan Gao, Ann-Lii Cheng, Lee X Li, Natalie Parent, Ganessan Kichenadasse, Christos S Karapetis, Andrew Rowland, Ashley M Hopkins, Michael J Sorich

Table of Contents

Supplementary Table 1 2
Supplementary Table 2 3
Supplementary Figure 1 4
Supplementary Figure 2 5

Supplementary Table 1. Demographic features of the included patient population

Characteristic	Overall N = 408[1]	Atezo+Bev N = 272[1]	Sorafenib N = 136[1]	p[2]
Age (years)	66 (59, 72)	66 (59, 72)	67 (61, 72)	0.3
Sex				0.8
Female	75 (18%)	51 (19%)	24 (18%)	
Male	333 (82%)	221 (81%)	112 (82%)	
Region				0.13
Asia	198 (49%)	122 (45%)	76 (56%)	
Europe	124 (30%)	86 (32%)	38 (28%)	
North America	77 (19%)	56 (21%)	21 (15%)	
Oceania	9 (2%)	8 (3%)	1 (1%)	
ECOG performance status score				>0.9
0	265 (65%)	177 (65%)	88 (65%)	
1	143 (35%)	95 (35%)	48 (35%)	
Child-Pugh classification				0.7
Grade A	405 (99%)	269 (99%)	136 (100%)	
Grade B	1 (0.2%)	1 (0.4%)	0 (0%)	
Missing	2 (0.5%)	2 (0.7%)	0 (0%)	
Barcelona Clinic liver cancer stage				0.9
A	11 (3%)	7 (3%)	4 (3.0%)	
B	65 (16%)	42 (15%)	23 (17%)	
C	332 (81%)	223 (82%)	109 (80%)	
Presence of macrovascular invasion and/or extrahepatic spread at study entry	301 (74%)	205 (75%)	96 (71%)	0.3
Varices	115 (28%)	78 (29%)	37 (27%)	0.8
Prior local therapy	189 (46%)	122 (45%)	67 (49%)	0.4
Cause of hepatocellular carcinoma				>0.9
Hepatitis B	160 (39%)	106 (39%)	54 (40%)	
Hepatitis C	100 (25%)	68 (25%)	32 (24%)	
Non-viral	148 (36%)	98 (36%)	50 (37%)	
Alcohol consumption				0.4
Current	69 (17%)	46 (17%)	23 (17%)	
Never	132 (32%)	82 (30%)	50 (37%)	
Previous	207 (51%)	144 (53%)	63 (46%)	
Smoking history				0.7
Current	73 (18%)	47 (17%)	26 (19%)	
Never	144 (35%)	100 (37%)	44 (32%)	
Previous	191 (47%)	125 (46%)	66 (49%)	
Metastatic sites				0.2
0	216 (53%)	140 (51%)	76 (56%)	
1	159 (39%)	114 (42%)	45 (33%)	
2	31 (7.5%)	17 (6%)	14 (10%)	
3	2 (0.5%)	1 (5%)	1 (1%)	

[1] Median (IQR); n (%)

[2] Wilcoxon rank sum test; Pearson's Chi-squared test; Fisher's exact test

[3] Atezo+Bev: Atezolizumab plus bevacizumab

Supplementary Table 2. Baseline information on biomarkers by treatment group

	Total population				TF HPD			
**Predictors**	**Overall** **N = 408**[1]	**Atezo+Bev**[3] **N = 272**[1]	**Sorafenib** **N = 136**[1]	***p***[2]	**Overall** **N = 55**[1]	**Atezo+Bev**[3] **N = 23**[1]	**Sorafenib** **N = 32**[1]	***p***[2]
PLR	129 (95, 192)	130 (97, 197)	128 (91, 184)	0.4	169 (113, 288)	182 (149, 294)	154 (93, 237)	0.08
$CRP_{mg/L}$	5.00 (2.00, 18.00)	5.00 (2.00, 18.00)	5.00 (2.00, 1.00)	0.8	8.00 (3.00, 36.00)	12.00 (3.00, 29.00)	7.00 (3.00, 41.00)	0.70
LMR	2.58 (1.79, 3.60)	2.85 (1.78, 3.61)	2.56 (1.82, 3.57)	0.9	2.19 (1.47, 3.13)	2.06 (1.38, 2.32)	2.33 (1.76, 3.43)	0.09
NLR	2.82 (1.96, 4.20)	2.92 (1.98, 4.20)	1.60 (1.95, 4.18)	0.5	3.58 (2.30, 5.97)	4.08 (2.69, 6.66)	3.11 (2.20, 5.47)	0.12
$\log(AFP)_{ug/L}$	3.99 (1.79, 7.42)	3.66 (1.78, 7.68)	4.73 (1.89, 7.37)	0.6	6.40 (3.50, 9.30)	8.60 (5.20, 9.50)	5.90 (2.90, 7.90)	0.08
ALBI								
Grade 1	209 (51%)	143 (53%)	66 (49%)	0.4	23 (42%)	15 (47%)	8 (35%)	0.4
Grade 2	199 (49%)	129 (47%)	70 (51%)		32 (58%)	17 (53%)	15 (65%)	

AFP = alpha-fetoprotein; ALBI = Albumin-Bilirubin Grade; CRP = c-reactive protein; LMR = lymphocyte to monocyte ratio; NLR = Neutrophil-to-lymphocyte ratio; PLR = platelet to lymphocyte ratio

[1] Median (IQR); n (%)

[2] Wilcoxon rank sum test; Pearson's Chi-squared test; Fisher's exact test

[3] Atezo+Bev: Atezolizumab plus bevacizumab

Supplementary Figure 1. Inclusion and exclusion criteria for the study population

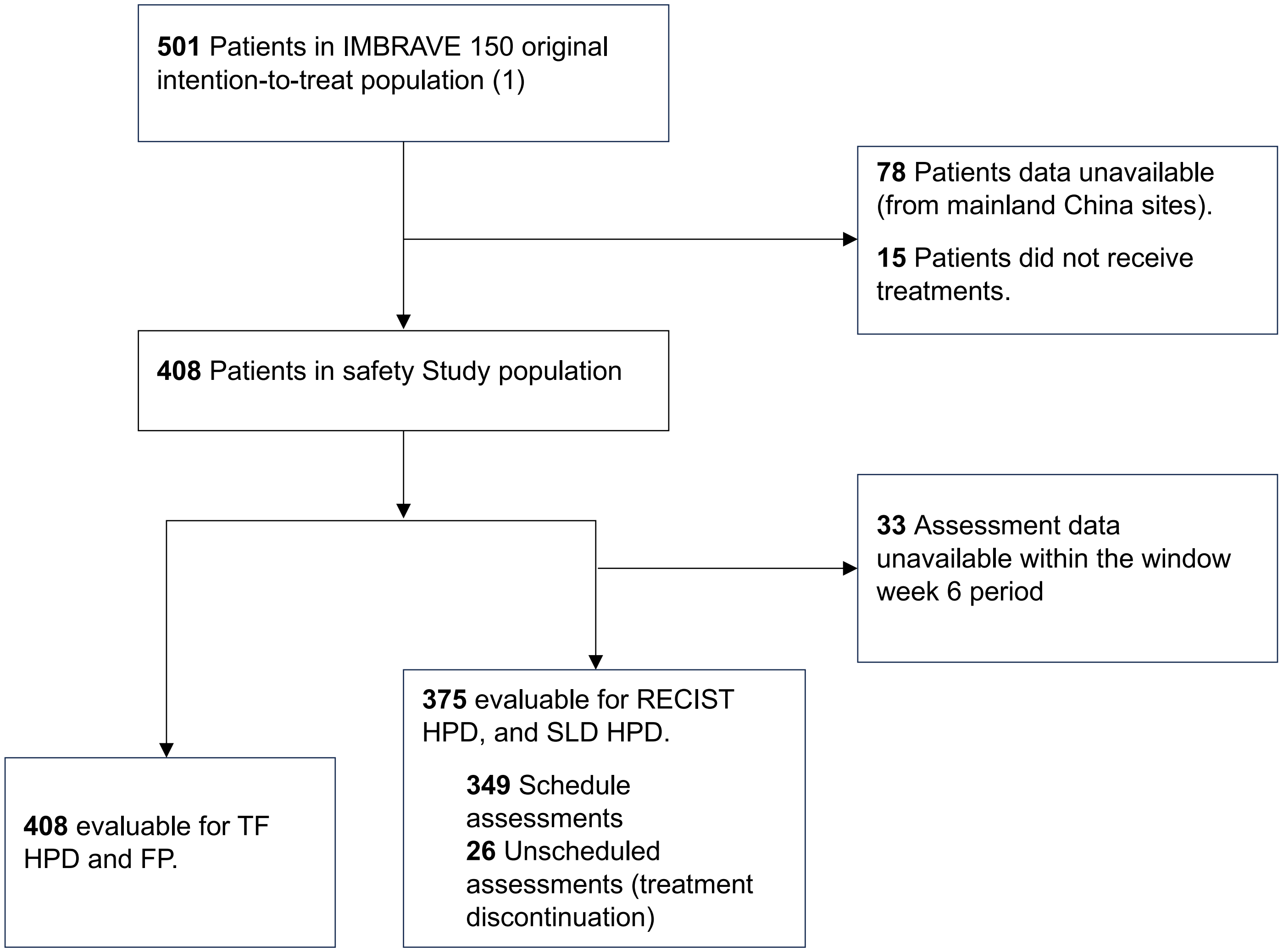

1. Finn RS, Qin S, Ikeda M, Galle PR, Ducreux M, Kim T-Y, et al. Atezolizumab plus Bevacizumab in Unresectable Hepatocellular Carcinoma. New England Journal of Medicine. 2020;382(20):1894-905

Supplementary Figure 2. Percent change of log(NLR) and log(AFP) from baseline to TF HPD onset/week 8 assessment

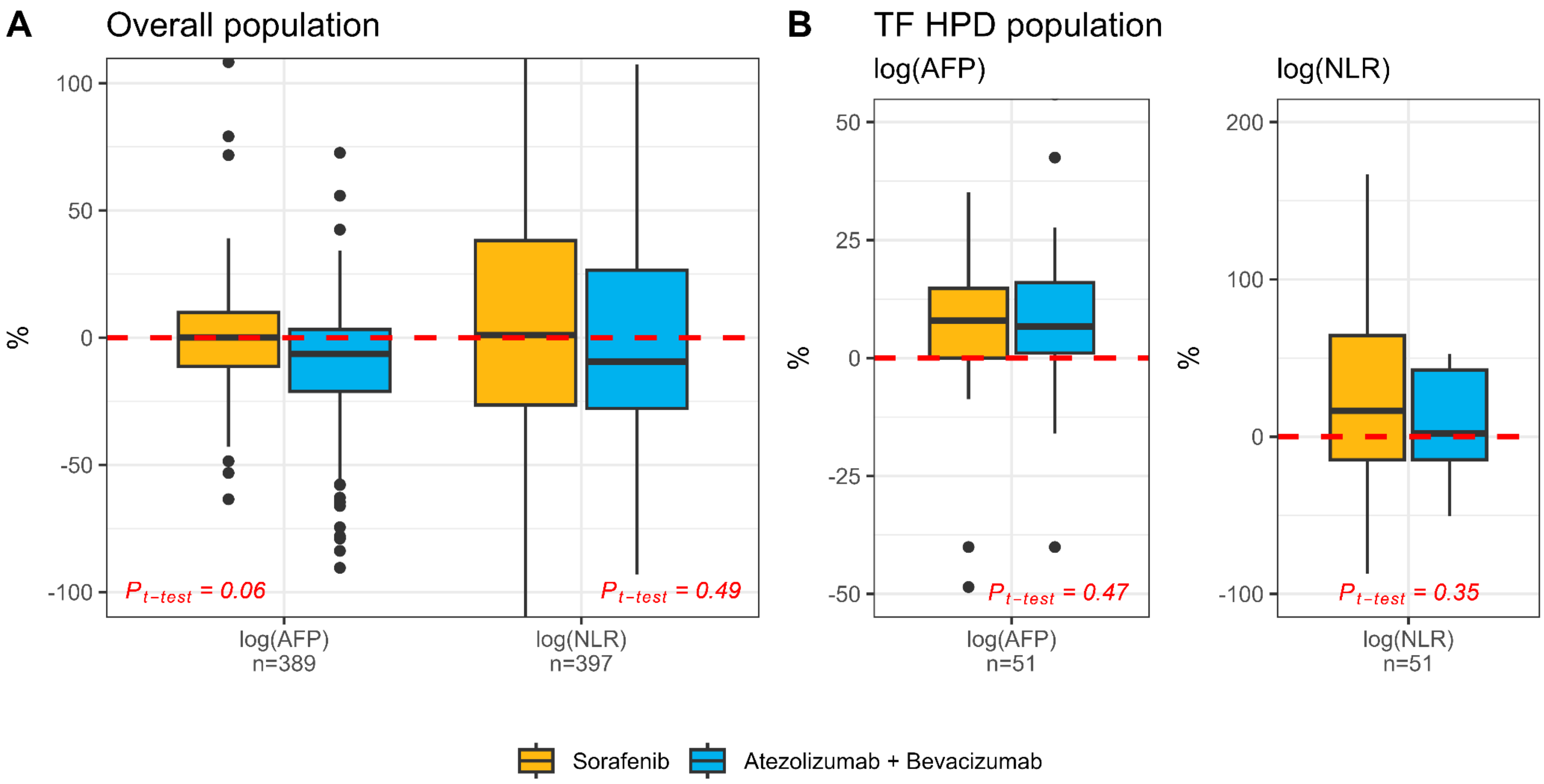

Supplement: Supplementary file 1 — DATA S1. Supporting Information. [file IJC-157-336-s001.pdf]
